# Gender role attitudes and fertility intentions: the mediating role of parental sacrifice and the moderating role of subjective well-being

**DOI:** 10.1186/s40359-024-01896-2

**Published:** 2024-07-18

**Authors:** Jiamiao Zhang, Gongxing Chen, Yingying Hu, Yuan Gao

**Affiliations:** 1 School of Psychology, Key Laboratory of Human Development and Mental Health of Hubei Province, Central China Normal University, Wuhan, 430079 China; 2 Center for Mental Health, Guangxi Vocational College of Water Resources and Electric Power, No. 99 Changgang Road, Xingning District, Nanning City, 530023 Guangxi Province China; 3 College of Education for the Future, Beijing Normal University at Zhuhai, Zhuhai, 519087 China; 4 Center for Mental Health, Beijing University of Agriculture, Beijing, 102206 China

**Keywords:** Gender role attitudes, Parental sacrifice, Subjective well-being, Fertility intentions, Family

## Abstract

Gender role attitudes have been shown to play a critical role in individuals’ fertility intentions. However, the underlying mechanism is unclear. The present study examined whether parental sacrifice mediates the relationship between gender role attitudes and fertility intentions, and whether subjective well-being plays a moderating role. A sample of 446 Chinese adults aged 18 to 45 (M_age_ = 32.78, SD_age_ = 5.63, 60.93% female) completed the Gender Role Attitude Scale, Parental Sacrifice Scale, Index of Well-Being, and Fertility Attitude Scale. Multiple regression analyses showed that traditional gender role attitudes positively predicted fertility intentions, while egalitarian gender role attitudes negatively predicted fertility intentions. Moreover, parental sacrifice was found to partially mediate the relationship between gender role attitudes and fertility intentions. Additionally, subjective well-being was identified as a moderator of the mediating effect of parental sacrifice. Specifically, for individuals with low subjective well-being, parental sacrifice played a partially mediating role. However, for individuals with high subjective well-being, the mediating effect of parental sacrifice was not significant, and gender role attitudes directly influenced fertility intentions. This study adds to our understanding of the connection between gender role attitudes and fertility intentions of adults, providing important information for policymakers and professionals aiming to promote fertility intentions.

## Introduction

The aging of the population is a crucial factor that significantly impacts social development. At the core of the aging population issue lies the notable phenomenon of a declining fertility rate [1, 2]. According to the results of the seventh national census released by the National Bureau of Statistics of China in May 2021, the country’s fertility rate has further decreased, reaching an all-time low of 1.3 [3]. Previous studies have demonstrated that low fertility intentions play a pivotal role in driving these declining fertility rates [4, 5]. Fertility intentions encompass individuals’ aspirations regarding parenthood, including their decision to have or not have children, as well as their desired number of children throughout their lifetime [6, 7].

Gender roles play a critical role in determining individuals’ fertility intentions [8–10]. Gender role attitudes refer to an individual’s perceptions and beliefs about gender roles and expectations, which are influenced by their experiences and exposure to social roles as a man or woman [11]. The underlying mechanism of how gender role attitudes predict fertility intentions is unclear. In East Asian households, especially in China, parents tend to display a careful and conscientious approach to fulfilling their children’s needs, often exceeding expectations in their sacrifices and contributions towards their children’s upbringing [12]. This includes giving up personal items of significance and dedicating themselves fully to fulfilling the child’s needs [13]. Parental sacrifice, as one of the fundamental concepts in Chinese family values, has become a widespread phenomenon in modern society [14]. It refers specifically to parents relinquishing their personal needs to address the developmental needs of their children [14]. We infer that parental sacrifice may affect the willingness to have children in traditional cultural contexts. Therefore, this study aims to investigate the predictive effect of gender role attitudes on fertility intentions and the mediating role of parental sacrifice.

Parental sacrifice can be viewed as a depletion of resources, such as time, money, and energy [14]. According to the Conservation of Resources Theory (COR), when an individual experiences a depletion of resources, it triggers tension and stress reactions that may be alleviated by acquiring additional resources [15]. Subjective well-being typically refers to an individual’s overall evaluation of their quality of life based on self-defined criteria [16]. It serves as a comprehensive psychological index for measuring an individual’s quality of life [16]. Individuals with a strong sense of subjective well-being perceive themselves as having a higher quality of life, experiencing more positive emotions, and fewer negative emotions [17]. They are also likely to view the loss of resources from parenting in a positive light, which can help decrease tension and stress. Therefore, the present study would also test the moderating effect of subjective well-being to provide a theoretical and practical foundation for addressing societal issues such as declining birth rates and aging populations.

## Theoretical framework

### Gender role attitudes and fertility intentions

In modern society, gender role attitudes encompass two distinct orientations: The first is the traditional perspective, which emphasizes upholding a division of labor between men and women and differentiating treatment based on physiological differences; the second is the modern perspective, which emphasizes equality between genders in education, employment, and political status [18]. It advocates for evaluating individuals based on their abilities and accomplishments rather than gender [19].

Using the Theory of Planned Behavior (TPB) as a conceptual framework, the study examined the relationship between gender role attitudes and fertility intentions. The Theory of Planned Behavior posits that an individual’s behavioral intentions play a vital role in predicting actual behavior [20–23]. These intentions are influenced by three main factors: behavioral attitudes, subjective norms, and perceived behavioral control [20]. Background factors, such as personal and sociocultural factors (e.g., personality, intelligence, experience, age, gender, and cultural background), indirectly influence attitudes, subjective norms, and perceived behavioral control by shaping the beliefs individuals hold, which may further impact behavioral intentions and behaviors [24]. Gender role attitudes encompass individuals’ expectations and beliefs regarding male and female reproductive roles, which can be traditional or modern, and thus shape individuals’ positive or negative evaluations of reproductive behavior [9, 25, 26]. Therefore, in the TPB, gender role attitudes may serve as background factors that influence an individual’s fertility attitudes through fertility beliefs, and then impact fertility intentions [24].

Some empirical studies have shown that individuals with more egalitarian gender role attitudes exhibit lower fertility intentions [8, 10]. Since the mid-20th century, with the development of industrialization, individuals’ gender role attitudes have become more and more egalitarian [9]. This gender role equality is more evident in the public sphere, that is, women have more widely joined the labor market [9]. The shift implies that women need to balance their career development in the public sphere and household chores in the private sphere, and the conflict between work and family may be one of the reasons for a weak desire to have children. Research has also indicated that an individual’s fertility intentions are shaped not only by their personal attitudes but also by the attitudes of their significant other [23]. The decision to have children is commonly a collaborative one made by both partners [27]. Therefore, the influence of men’s gender role attitudes on fertility intentions is also crucial. Research has found that men with egalitarian gender role attitudes are less likely to have children [28–30]. Therefore, the first hypothesis is proposed.

#### H1:

Traditional gender role attitudes would positively predict fertility intentions, while egalitarian gender role attitudes would negatively predict fertility intentions.

### Gender role attitudes, parental sacrifice, and fertility intentions

A cultural perspective of Role Theory provided the conceptual framework for examining the relationship between gender role attitudes and parental sacrifice [31, 32]. Parental sacrifice reflects parents’ contribution to their children’s education, referring to the process in which parents give up their personal needs for their children’s education [14]. Parental sacrifice has always been regarded as a core characteristic of Chinese family values. There is a dynamic interplay between the social and cultural environment and the household environment. Culture has a critical impact on shaping the beliefs, attitudes, and behaviors of parents towards their childrearing practices [32]. Influenced by traditional Chinese culture, such as the saying “Wang zi cheng long, wang nü cheng feng” which means the expectations for children to become accomplished individuals. Parents place great emphasis on their children’s education and are even willing to make concessions to meet their educational needs [14].

Based on a cultural perspective of Role Theory [31, 32], fathers with traditional gender role attitudes are expected to earn income and discipline their children. They are responsible for mobilizing resources for the family and child development, as the saying goes, “If a child is not taught, it is the father’s fault” [33, 34]. Therefore, men with traditional gender role attitudes are more likely to make more parental sacrifices in educating their children. Mothers, on the other hand, perform the expressive function, participating in household management and child care, and providing emotional support for their children [32]. Therefore, traditional gender role attitudes may predict higher levels of parental sacrifice, while the trend toward gender role attitude equality may predict lower levels of parental sacrifice. The Economic Theory of Fertility Decline posits that parental fertility choices are the result of a rational trade-off between the benefits of children and the costs of having them [35]. When the costs of raising a child exceed the benefits that the child brings, the family’s fertility intentions decrease [35]. Conversely, when the benefits of having children outweigh the costs, the family’s fertility intentions increase [35]. Parental sacrifice is akin to parental investment [36]. Parents who are willing to make more sacrifices may place a higher value on the potential psychological and social rewards of such costs, such as the warmth of the family, the success of their children, and their elevated social status, which enhances their willingness to have children. This study proposes the second hypothesis.

#### H2:

Parental sacrifice would mediate the relationship between gender role attitudes and fertility intentions.

### Gender role attitudes, parental sacrifice, subjective well-being, and fertility intentions

Equality is a manifestation of human civilization’s progress, and the trend toward gender role equality is irreversible. Our research aims to explore how to regulate the potential decrease in fertility intentions that may result from this trend. Researchers have noticed the role of subjective well-being (SWB), a comprehensive psychological indicator reflecting an individual’s quality of life and social functioning and adaptation [37]. According to the Broaden-and-Build Theory of Positive Emotions, individuals’ subjective well-being reflects the experience of positive emotions, which help broaden individuals’ thought-action repertoires at the moment [38]. This broadening effect, in turn, builds lasting personal resources, including physical, intellectual resources, social, and psychological resources [38]. The COR theory posits that when resources are scarce, individuals tend to proactively seek alternative resources to cope with environmental pressure [15]. The act of parents making sacrifices may result in a reduction of available resources [14], and the presence of subjective well-being, which is associated with valuable resources [38, 39], has the potential to alleviate this strain [40]. Should increased subjective well-being lessen the burden of parental sacrifices, individuals may not feel the need to counteract this strain by opting to have fewer children. Some researchers suggested that higher subjective well-being could reduce the adverse effect of job uncertainty on fertility intentions [4]. Factors such as social and cultural context [41], personal income [42], and self-perception [43] are related to subjective well-being and are also relevant to fertility and parenting. Therefore, the present study proposes the third hypothesis.

#### H3:

H3: Subjective well-being would moderate the effect of parental sacrifice on fertility intentions.

**Fig. 1:**
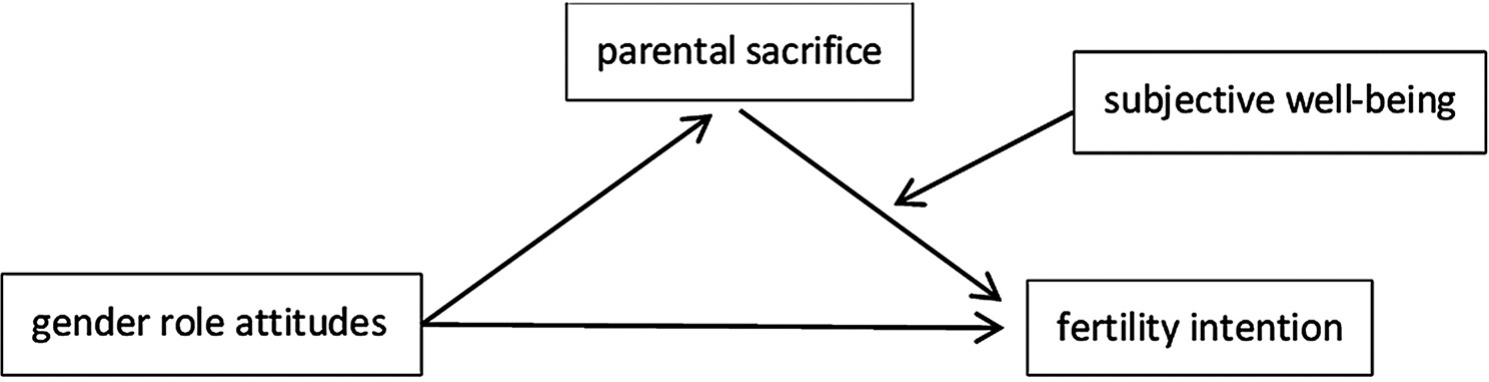
A moderated mediation model

In summary, we constructed a moderated mediation model (Fig. 1) to test the mediating role of parental sacrifice between gender role attitudes and fertility intentions, as well as the moderating role of subjective well-being.

## Methods

### Participants and procedures

This study was approved by the Research Ethics Committee of Guangxi Vocational College of Water Resources and Electric Power. Before collecting data, we provided participants with a written informed consent form. The questionnaires were distributed in high, medium, and low economic development areas of China using a stratified sampling method. The participants were in three provinces: Guangdong, Jiangxi, and Guangxi. The data collection was conducted through a professional survey company in China (https://www.wjx.cn/) and it took approximately 20 min to complete the questionnaires. Invalid data was characterized by a subject consistently providing identical responses to all items on the scales. A total of 446 questionnaires were distributed, and 430 valid questionnaires were collected (96.41%). The sample consisted of 262 females (60.93%) and 168 males (39.07%), aged 18 to 45 years old (M = 32.78, SD = 5.63). 149 individuals (65.3%) lived in rural areas, while 281 individuals (34.7%) lived in urban areas. Of the total sample, 145 individuals (33.7%) did not have children, 162 individuals (37.7%) had one child, 118 individuals (27.4%) had two children, and 5 individuals (1.2%) had three or more children.

### Measures

#### Gender role attitudes

The Gender Role Attitude Scale developed by the Renmin University of China in the 2015 China General Social Survey [19] was used to define gender role attitudes along two dimensions: modernity and traditionality, with a focus on the equalization of gender role attitudes. Specifically, the scale included five dimensions: family and career (e.g., “Men should focus on their careers, while women should focus on family.“), ability traits (e.g., “Men are naturally superior to women in ability.“), self-worth (e.g., “It is better to marry well than to do well.“), labor and employment (e.g., “In times of economic downturn, women should be laid off first.“), and household division of labor (e.g., “Husbands and wives should share household chores equally.“). A 5-point Likert scale (1 = strongly disagree, 5 = strongly agree) was used, with higher scores indicating more traditional gender role attitudes. The Cronbach’s alpha coefficient of the scale in this study was 0.74.

#### Parental sacrifice

The Parental Sacrifice Scale developed by Leung et al. was used [14], which consists of 5 dimensions and 23 items. The 5 dimensions are: seeking family resources (e.g., “I cut down on my expenses to meet my children’s educational needs.“), spending time (e.g., “During exam periods, I try to stay at home with my children as much as possible.“), adjusting schedules (e.g., “My daily routines are adjusted according to my children’s educational needs.“), sacrificing lifestyle (e.g., “I give up my social life for the sake of my children’s education.“), and concealing anxiety (e.g., “I hide family problems from my children to avoid affecting their studies.“). The scale was scored on a 6-point Likert scale (1 = completely disagree, 6 = completely agree). A higher score indicates a greater level of parental sacrifice. In this study, the Cronbach’s alpha coefficient of the scale was 0.94.

#### Subjective well-being

The Index of Well-Being developed by Campbell et al. was used in this study [44]. The scale consists of two dimensions: the general affective index and life satisfaction. The former is composed of eight items with a weight of 1 (e.g., “What is your emotional experience of life? 1 = hopeless, 7 = hopeful”), and the latter consists of only one item with a weight of 1.1 (i.e., “How satisfied are you with life in general?” 1 = extremely dissatisfied, 7 = extremely satisfied). A 7-point Likert scale was used, with higher total scores indicating greater subjective well-being. The retest reliability of the total scale is 0.85 in the Chinese sample [45]. The Cronbach’s alpha coefficient for the scale in this study was 0.94.

#### Fertility intentions

The Fertility Attitude Scale developed by Arnocky et al. was translated into Chinese and utilized to assess fertility intentions [6], employing a 5-point Likert scale (e.g., “My having children is important for my entire family”, 1 = completely disagree, 5 = completely agree). It comprises two distinct subscales: Pro-reproductive and Anti-reproductive attitudes, with Cronbach’s alpha coefficient of 0.70 and 0.75, respectively. The fertility attitude score is calculated by subtracting the score of the anti-fertility attitude scale from that of the pro-fertility attitude scale. The Cronbach’s alpha coefficient for the scale in this study was 0.66.

#### Demographic measures

Demographic information, including age, gender, household registration, and number of existing children, was collected. These variables were treated as covariates in the data analysis. Gender was dummy coded as 1 = male, 0 = female. Household registration was dummy coded as 1 = urban, 0 = rural. The number of existing children was dummy coded as 1 = with 2 or more children, 0 = with 0 or 1 child.

### Data analysis

The data was managed and analyzed using SPSS 22.0 and Mplus 8.3. SPSS 22.0 was primarily used for conducting common method variance tests, descriptive statistics, correlation analyses, and independent sample t-tests. On the other hand, Mplus 8.3 was employed to analyze the moderated mediation model using conditional process analysis, which involved ordinary least squares (OLS) regression with a bootstrapping technique [46]. The initial step of the analysis involved standardizing the data, followed by multiplication of the z-scores of parental sacrifice and subjective well-being to obtain interaction term scores. A bias-corrected percentile Bootstrap method with 3000 replicate samples was utilized for testing, and 95% confidence intervals were estimated. Control variables included gender, household registration, and the number of existing children.

## Results

### Common method variance tests

This research employed the questionnaire methodology, potentially susceptible to common method variance. The research questionnaire underwent assessment for common method variance using Harman’s single-factor test before statistical analysis. Factor analysis of all tested items revealed 9 factors with eigenvalues exceeding 1 in the unrotated results. These factors collectively explained 65.16% of the total variance. The initial factor explained 23.01% of the total variance, falling below the critical threshold of 40% [47]. These findings suggest that there was no significant common method variance present in this study.

### Descriptive statistics and the correlation among the studied variables

The findings of this study present the descriptive statistics and correlation analysis results for each variable, as displayed in Table 1. Fertility intentions were positively correlated with household registration (*r* = 0.16, *p* < 0.01), the number of existing children (*r* = 0.10, *p* < 0.05), parental sacrifice (*r* = 0.28, *p* < 0.01), and gender role attitudes (*r* = 0.46, *p* < 0.01), while being negatively correlated with gender (*r* = -0.39, *p* < 0.01). Moreover, subjective well-being was found to have no significant correlation with fertility intentions (*r* = 0.08, *p* > 0.05). As a result, gender, household registration, and the number of existing children were employed as control variables in the subsequent statistical analysis due to their association with fertility intentions. The reliability coefficients expressed by Cronbach’s α ranged from 0.66 (fertility intentions) to 0.94 (subjective well-being), indicating satisfactory internal reliability for all variables. An independent sample t-test was conducted to examine the differences in fertility intentions based on demographic variables. Results showed that men had significantly higher fertility intentions than women (*t* = 8.77, *p* < 0.001). Additionally, individuals living in rural areas had significantly higher fertility intentions than those living in urban areas (*t* = -3.42, *p* < 0.01). Finally, individuals who have two or more children had significantly higher fertility intentions than those who have no children or only one child (*t* = -1.99, *p* < 0.05).

**Table 1:** Descriptions, correlation matrix, and reliabilities for each variable (*n* = 430)

	M ± SD	1	2	3	4	5	6	7	8
1. Age	32.78 ± 5.63	1							
2. Gender	0.61 ± 0.49	0.22^**^	1						
3. HR	0.35 ± 0.48	0.48^**^	0.22^**^	1					
4. NEC	0.29 ± 0.45	0.39^**^	0.09	-0.20^**^	1				
5. SWB	1.15 ± 0.25	0.13^**^	0.05	-0.07	0.20^**^	**(0.94)**			
6. PS	4.23 ± 0.88	0.07	-0.13^**^	0.06	0.04	0.06	**(0.94)**		
7. GRA	2.03 ± 0.79	-0.02	-0.43^**^	0.14^**^	0.05	-0.07	0.23^**^	**(0.74)**	
8. FI	0.41 ± 0.61	0.06	-0.39^**^	0.16^**^	0.10^*^	0.08	0.28^**^	0.46^**^	**(0.66)**

*Note* Gender Role Attitudes (GRA), Parental Sacrifice (PS), Subjective Well-Being (SWB), Fertility Intentions (FI), Household Registration (HR), and Number of Existing Children (NEC). Gender, HR, and NEC were dummy variables. Gender: male = 1, female = 0; HR: urban = 1, rural = 0. NEC: With 2 or more children = 1, With 0 or 1 child = 0. ^*^p < 0.05, ^**^p < 0.01, ^***^p < 0.001. Cronbach’s alphas are in the diagonal in bold

### Moderated mediation model test

A moderated mediation analysis was used to test whether the mediation effect of parental sacrifice on the relationship between gender role attitudes and fertility intentions was influenced by subjective well-being. According to Muller et al. [48], the test of the moderated mediation model involves constructing three equations as follows.

$$\:\text{E}\text{q}\text{u}\text{a}\text{t}\text{i}\text{o}\text{n}\:1:Y={\beta\:}_{10}+{\beta\:}_{11}X+{\beta\:}_{12}{M}_{o}+{\beta\:}_{13}X{M}_{o}+{\epsilon\:}_{1}$$

$$\:\text{E}\text{q}\text{u}\text{a}\text{t}\text{i}\text{o}\text{n}\:2:\:{M}_{e}={\beta\:}_{20}+{\beta\:}_{21}X+{\beta\:}_{22}{M}_{o}+{\beta\:}_{23}X{M}_{o}+{\epsilon\:}_{2}$$

$$\:\text{E}\text{q}\text{u}\text{a}\text{t}\text{i}\text{o}\text{n}\:3:\text{Y}={\beta\:}_{30}+{\beta\:}_{31}X+{\beta\:}_{32}{M}_{o}+{\beta\:}_{33}X{M}_{o}+$$

$${\beta\:}_{34}{M}_{e}+{\beta\:}_{35}{M}_{e}{M}_{o}+{\epsilon\:}_{3}$$

The moderated mediation effect holds subject to the following two conditions: First, in Eq. 1, *β*_11_ is significant, while *β*_13_ is not; Second, in Eqs. 2 and 3, either *β*_23_ or *β*_35_ is significant, or both.*β*_23_ and *β*_34_ are significant simultaneously, or *β*_35_ and *β*_21_ are significant simultaneously. In the present study, X represents gender role attitudes, *M*_*e*_ represents parental sacrifice, *M*_*o*_ represents subjective well-being, and *Y* represents fertility intentions.

Data were standardized before the analysis. Gender, household registration, and number of existing children were controlled. The results are shown in Table 2. Equation 1 showed that gender role attitudes positively and significantly predicted fertility intentions (*β*_11_ = 0.36, *t* = 7.59, *p* < 0.001), but the interaction term of gender role attitudes and subjective well-being was not statistically significant on fertility intentions (*β*_13_ = -0.03, *t* = -0.53, *p* > 0.05). In support of Hypothesis 1, the results indicated that more traditional gender role attitudes may predict stronger fertility intentions, while more equal gender role attitudes may predict weaker fertility intentions. The first condition for a moderated mediation model was met. In Eqs. 2 and 3, gender role attitudes positively and significantly predicted parental sacrifice (*β*_21_ = 0.22, *t* = 4.03, *p* < 0.001), parental sacrifice positively and significantly predicted fertility intentions (*β*_34_ = 0.16, *t* = 3.55, *p* < 0.001), and the interaction term of parental sacrifice and subjective well-being significantly predicted fertility intentions (*β*_35_ = -0.10, *t* = -2.15, *p* < 0.05). The second condition for a moderated mediation model was met as both *β*_21_ and *β*_35_ were significant simultaneously. The results indicated a moderated mediation model between gender role attitudes and fertility intentions. Parental sacrifice was a mediator, while subjective well-being moderated the relationship between parental sacrifice and fertility intentions. Hypothesis 2 and 3 were supported.

**Table 2:** Tests for mediating effects with moderation

	Equation 1 FI			Equation 2 PS			Equation 3 FI		
	β	se	t	β	se	t	β	se	t
GRA	0.36	0.05	7.59^***^	0.22	0.06	4.03^***^	0.32	0.05	6.77^***^
SWB	0.11	0.05	2.07^*^	0.07	0.06	1.27	0.10	0.05	2.24^*^
GRA * SWB	-0.03	0.05	-0.53	-0.03	0.05	-0.61	0.01	0.05	0.23
PS							0.16	0.04	3.55^***^
PS * SWB							-0.10	0.04	-2.15^*^
Gender	-0.48	0.10	-4.63^***^	-0.07	0.11	-0.62	-0.47	0.10	-4.74^***^
HR	0.20	0.10	2.06^*^	0.07	0.10	0.71	0.20	0.09	2.16^*^
NEC	0.21	0.09	2.39^*^	0.05	0.10	0.53	0.19	0.09	2.15^*^
R^2^	0.27			0.06			0.31		
F	26.08^***^			4.48^***^			23.64^***^		

*Note* Gender Role Attitudes (GRA), Parental Sacrifice (PS), Subjective Well-Being (SWB), Fertility Intentions (FI), Household Registration (HR), and Number of Existing Children (NEC). Gender, HR, and NEC were dummy variables. Gender: male = 1, female = 0; HR: urban = 1, rural = 0. NEC: With 2 or more children = 1, With 0 or 1 child = 0. ^*^p < 0.05, ^**^p < 0.01, ^***^p < 0.001

**Fig. 2:**
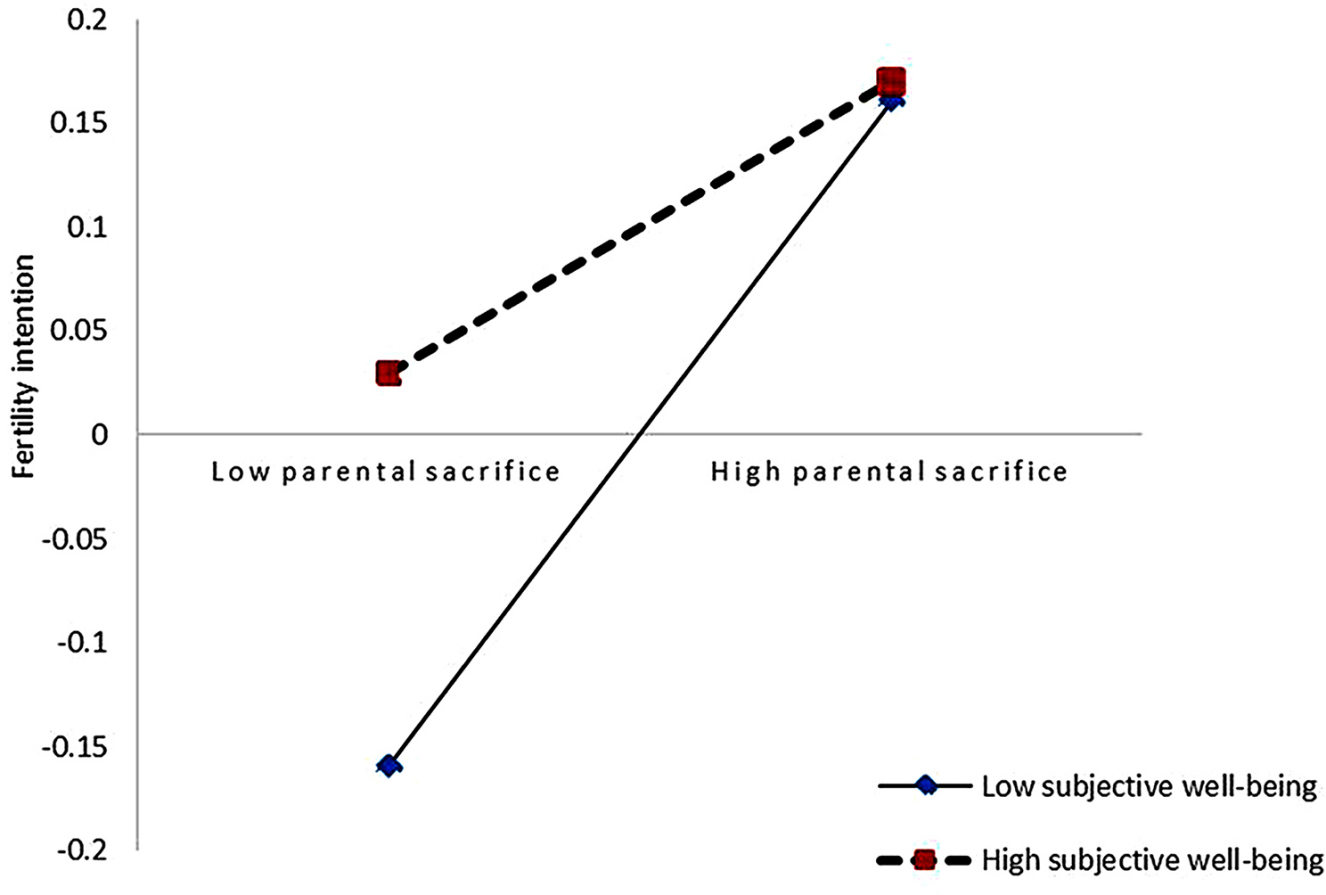
The moderating role of Subjective Well-Being in the relationship between Parental Sacrifice and Fertility Intentions

Based on the mean ± 1 standard deviation of subjective well-being, the mediating effect of parental sacrifice between gender role attitudes and fertility intentions and their 95% CI were divided into two levels. The mediating effect with moderation holds as shown in Table 3. To further analyze the substance of the moderating effect, a simple slope test was conducted, and a schematic diagram of the moderating effect was drawn (Fig. 2). The findings suggested that parental sacrifice had a positive association with fertility intentions when individuals had low levels of subjective well-being (simple slope = 0.052, 95% CI [0.022, 0.099]), but the effect was not significant when subjective well-being was high (simple slope = 0.013, 95% CI [-0.010, 0.044]). Overall, higher levels of parental sacrifice were associated with stronger fertility intentions. Specifically, for those with low parental sacrifice, higher levels of subjective well-being were linked to stronger fertility intentions, implying that high subjective well-being attenuates the impact of parental sacrifice on fertility intentions.

**Table 3:** Mediating effects at different levels of subjective well-being

SWB	Path a X-> M	Path b M-> Y	Indirect Effect	BootSE	BootLLCI	BootULCI
Mean-SD	0.212	0.247	0.052	0.019	0.022	0.099
Mean + SD	0.212	0.063	0.013	0.014	-0.010	0.044
DIFF	0	0.184	0.039	0.020	0.007	0.088

*Note* Subjective Well-Being (SWB), 95% BootstrapCI does not include zero, then the effect is significant

## Discussion

The low fertility rate is becoming a key concern in social development. Although studies have examined the relationship between gender role attitudes and fertility intentions, the underlying mechanism remains unclear. Based on the Theory of Planned Behavior (TPB) and the Conservation of Resources Theory (COR), this study constructed a moderated mediation model to investigate the predictive effect of gender role attitudes on fertility intentions and its underlying mechanism. Parental sacrifice was found to play a mediating role in the relationship between gender role attitudes and fertility intentions, and subjective well-being moderated the predictive relationship between parental sacrifice and fertility intentions. Specifically, the equalization of gender role attitudes was likely to reduce adults’ parental sacrifice, which may resulted in lower fertility intentions. High subjective well-being may enhance the fertility intentions of adults with low parental sacrifice. This study is the first to test the mediating role of parental sacrifice between gender role attitudes and fertility intentions, as well as the moderating role of subjective well-being. These results are important because they provide theoretical and empirical evidence for addressing the issue of low fertility.

### The relationship between gender role attitudes and fertility intentions

Consistent with previous studies, the present study found that adults with more traditional gender role attitudes were more likely to have children, while those with more equal gender role attitudes were less likely to do so [8, 10]. The results provide support for the Theory of Planned Behavior. Adults’ perceptions and beliefs about the roles of men and women in society, culture, and the family as attitudes can predict their willingness to have children.

In the traditional gender role attitudes, the deeply rooted cultural belief of “men as breadwinners and women as homemakers” has significant negative impacts on women’s labor participation, which leads to women’s economic status being controlled [49]. This generates economic insecurity, which in turn displays higher fertility intentions [50]. On the contrary, gender equality has gradually been accepted by modern society [9]. Women with more equal gender role attitudes can enhance their economic and social status through greater labor expectations, and having children is no longer the sole expression of their value [19]. Men are also increasingly inclined to recognize the work of wives and mothers and believe that they should help with household chores [30, 51]. Therefore, egalitarian men may exhibit less willingness to have children compared to their more traditional counterparts. Determining the decision of whether to have children, as well as the number of children to consider, is a multifaceted process that involves the perspectives of both partners and potentially the broader family unit [23, 52, 53].

### The mediating role of parental sacrifice

This research is the first to prove the important role of parental sacrifice in fertility intentions. The study found that parental sacrifice partially mediated the relationship between gender role attitudes and fertility intentions. Studying individuals’ parental sacrifice is crucial for understanding fertility intentions within a traditional cultural context.

In a traditional cultural context, parental sacrifice is highly valued. From the perspective of cultural role theory, traditionally, fathers tend to provide educational resources for their children, while mothers tend to provide emotional value [33]. Individuals with modern gender role attitudes advocate egalitarianism, which may not only be limited to gender role attitudes but also extend to child-parenting. This is manifested in the belief that parents and children are equal and that parents should not sacrifice themselves for the sake of their children’s growth. Therefore, adults with more egalitarian gender role attitudes tend to make fewer sacrifices in child-parenting. In support of the Economic Theory of Fertility Decline, the time, money, and effort involved in parental sacrifice are considered costs [35, 36]. Adults who were less willing to sacrifice in parenting reported lower fertility intentions because they viewed the costs of raising a child as outweighing the benefits that the child brings. Nevertheless, additional confirmation is required to determine if this outcome can be applied to different cultural contexts.

### The moderating role of subjective well-being

This study found that subjective well-being could moderate the effect of gender role attitudes on fertility intentions through the indirect effect of parental sacrifice. Specifically, subjective well-being could reduce the positive effect of parental sacrifice on adults’ fertility intentions when they are facing low levels of parental sacrifice. However, subjective well-being did not have an effect at high levels of parental sacrifice. Subjective well-being was more likely to enhance fertility intentions among individuals with low parental sacrifice.

The results of the correlation analysis in this study revealed no correlation between parental sacrifice and subjective well-being. However, subjective well-being was found to moderate the effect of parental sacrifice on fertility intentions. The reason may be related to different forms of well-being, which are discussed in the existing literature. One form of well-being is hedonistic well-being, and subjective well-being is viewed from a hedonic perspective [54–57]. It pertains to the notion that well-being involves feeling satisfied with one’s overall life assessment and experiencing more positive emotions while encountering fewer negative emotions [58–60]. Another form of well-being is eudaimonic well-being, where individuals strive to achieve self-actualization by working towards realizing their potential for positive development and advancement [56, 61]. Parental sacrifice is the process by which a parent voluntarily gives up their interests, comfort, and security for the sake of a higher purpose and the well-being of their child [3, 14, 36, 62]. According to the eudaimonic perspective, individuals strive to achieve their goals, which is closer to the idea of parental sacrifice. Therefore, parental sacrifice is more likely to be associated with eudaimonic well-being than subjective happiness, which requires further validation.

Consistent with the Broaden-and-Build Theory of Positive Emotions, one possible explanation for the moderating effect of subjective well-being is that individuals with high levels of subjective well-being exhibit greater openness and creativity in their thinking, enabling them to contemplate a broader array of possibilities and solutions [38]. Consequently, they demonstrate enhanced adaptability and flexibility when confronted with problems and challenges in child-rearing. For individuals who are less willing to sacrifice in parenting, high levels of subjective well-being could increase their fertility intentions. Another possible explanation is that individuals with high subjective well-being are more satisfied with life, experience more positive emotions, and fewer negative emotions, which could build positive psychological resources [38]. Consistent with COR Theory, these resources built by subjective well-being could make up for the loss of resources from parental sacrifice [14], bringing total resources into balance and relieving stress [63, 64]. Therefore, subjective well-being may cause individuals with low parental sacrifice to view parental sacrifice more positively and not feel the need to reduce stress by having fewer children. These are also consistent with research that proves individuals with high subjective well-being have more positive evaluations and attitudes towards social culture [41], personal income [42], and self-perception [43]. In other words, individuals with subjective well-being have a positive social function and adaptive states, which positively predict fertility intentions when at a low level of parental sacrifice.

### Significance and contribution

This research contributes to enhancing our understanding of the relationship between gender role attitudes and adults’ fertility intentions, providing valuable insights for policymakers and practitioners aiming to promote fertility intentions.

Theoretically, this study integrates the analysis of gender role attitudes, parental sacrifice, subjective well-being, and fertility intentions within a cohesive framework. This synthesis offers a nuanced perspective on the factors associated with fertility intentions. The research also emphasizes a strong link between egalitarian gender role attitudes and reduced fertility intentions, contributing to the discussion on how modern societal values may influence fertility behaviors.

Practically, the results have potential applied value for developing new strategies to promote fertility intentions. Parental sacrifice is an important mechanism linking gender role attitudes and adults’ fertility intentions. Therefore, practitioners may provide parents with more resources to help them parent their children and reduce the burden of parental sacrifice. Moreover, subjective well-being plays a role in adults who report a low level of parental sacrifice. Therefore, practitioners may consider enhancing the subjective well-being of individuals to provide them with more resources to cope with the challenges of child-rearing.

### Limitations and future research

Several limitations to this study must be acknowledged. First, although we theoretically identified gender role attitudes as a predictor variable for fertility intentions, we cannot infer a causal relationship between gender role attitudes and fertility intentions with this cross-sectional design. This can be tested in future longitudinal or experimental studies. Second, the variables in this study were assessed using self-reported data, but fortunately, we statistically tested for the small impact of common method bias. Future research could consider multiple data sources. Third, the sample size was small in terms of sampling, and although stratification was used, only regional stratification was considered. In the future, more stratification could be designed to enhance sample representativeness and increase the generalizability of the study. Finally, the process of making fertility decisions is complex and may be influenced by the partner [23, 65]. However, this study tested the fertility intentions of only one spouse and did not consider the influence of the partner’s gender role attitudes on fertility intentions. In the future, paired studies involving couples could be conducted.

## Conclusion

This study constructed a moderated mediation model to investigate the mechanisms and related factors of fertility intentions. This is the first study to show that equal gender role attitudes were negatively associated with fertility intentions, partially mediated by parental sacrifice, and moderated by subjective well-being. Specifically, high levels of subjective well-being could weaken the impact of parental sacrifice on fertility intentions, and increasing subjective well-being could enhance fertility intentions among individuals with low levels of parental sacrifice. This study adds to our understanding of the connection between gender role attitudes and fertility intentions of adults, providing important information for policymakers and professionals aiming to promote fertility intentions.

## Data Availability

The datasets used and/or analysed during this study are available from the corresponding author on reasonable request.
